# Recent advances in operative techniques for pancreatic surgery

**DOI:** 10.1515/iss-2025-0017

**Published:** 2025-09-18

**Authors:** Johanna Strotmann, Verena Tripke

**Affiliations:** Department of General and Visceral Surgery, St. Josef Hospital Bochum, Ruhr-University Bochum, Bochum, Germany; Department of General, Visceral and Transplantation Surgery, University Medical Center of the Johannes Gutenberg University, Mainz, Germany; Chirurgische Arbeitsgemeinschaft Junge Chirurgie CAJC, Deutsche Gesellschaft für Allgemein- und Viszeralchirurgie, Berlin, Germany

**Keywords:** pancreatic surgery, robotic surgery, pancreatic cancer, postoperative pancreatic fistula

## Abstract

**Introduction:**

Pancreatic resections are one of the most complex operations in visceral surgery, characterized by high perioperative morbidity and mortality. Continuous evaluation and adjustment of surgical techniques and approaches are required to improve the quality of surgery and outcomes in this highly vulnerable collective of patients.

**Content:**

In this short narrative review we will discuss exemplary three recent advances in pancreatic surgery.

**Summary and Outlook:**

A systematic literature search was performed using the PubMed database. The three discussed promising developments in pancreatic surgery are the triangle operation, a radical resection of lymphatic and nerve tissue in patients with pancreatic cancer, autologous patches for covering skeletonized arteries after resection to reduce frequent postoperative complications, and minimal invasive surgical approaches, which offer potential benefits in reduced intraoperative blood loss, shorter hospital stay and lower rate of wound infections compared to open resections. These three new operative approaches represent promising advances in a demanding surgical field. However, further studies are needed to confirm the benefits of these techniques on patient outcomes.

## Introduction

The standard of care for curative treatment of pancreatic cancer is, depending on location, pancreatoduodenectomy (PD) or distal pancreatectomy (DP), especially the former is one of the most complex operations in visceral surgery [1]. Even in specialized centers PD is still associated with relatively high perioperative morbidity (up to 40 %) [2]. Surgical innovations aim to improve the procedural quality as well as outcomes in this vulnerable population.

Resection remains the only curative treatment option in these patients and apart from the high morbidity and mortality rates, the recurrence and overall survival rates remain short even after successful surgery [1]. Positive resection margins are associated with local recurrence; therefore, the TRIANGLE operation has been introduced as a more radical surgical approach [3]. During the TRIANGLE operation soft tissue between coeliac artery, superior mesenteric artery and mesoportal axis is additionally removed to improve recurrence free and overall survival [3].

Another challenge in pancreatic surgery are the associated severe and frequent complications including postoperative pancreatic fistula (POPF) and post-pancreatectomy hemorrhage (PPH) [2]. Both POPF and PPH are associated with significant mortality and increased hospital stay. Several surgical techniques utilizing autologous patches wrapped around the common hepatic artery and gastroduodenal artery stump have been developed to prevent POPF and reduce hospital stay and postoperative mortality [2].

To further improve the postoperative outcome of patients undergoing pancreatectomy, the minimally invasive approach was introduced to pancreatic surgery, offering potential benefits of reduced intraoperative blood loss, shorter hospital stay, and lower rate of wound infections compared to open resections [4], 5]. Robotic systems offer potential benefits in resection and reconstruction owing to their flexibility and improved range of motion [6]. However the learning curve to master this innovative system needs to be addressed [7].

This review aims to discuss these three recent and promising advances in terms of implementation, advantages and limitations in the following order: TRIANGLE operation, autologous patches and pancreatic robotic surgery.

## Methods

The PubMed database was used for the systematic literature search. The search terms included “postoperative pancreatic fistula”, “post-pancreatectomy hemorrhage”, “pancreatic surgery”, “robotic pancreatectomy”, “minimally invasive pancreatectomy”, “triangle operation, and “autologous patches”. To highlight recent advances in pancreatic surgery, the search focused mainly on articles published between 2021 and 2024.

Original articles, reviews, and meta-analyses were also included. Titles and abstracts were screened to identify relevant studies. As this short narrative review aimed to provide a concise overview of recent advances in pancreatic surgery, 10 relevant articles on three different topics were included and reviewed by both authors.

## Results

### Triangle operation

Pancreatic cancer is the 6th most common form of malignant disease and the most common cause of cancer-related death in Germany. Surgical resection remains the only curative treatment option available. However, recurrence rates are high, and overall survival even after successful surgery remains short [1]. Positive resection margins, especially in the retroperitoneum, are associated with local recurrence; thus, Hackert et al. introduced the TRIANGLE operation as a more radical surgical approach [3]. The standard of care features a locoregional lymph node dissection of the nodes adjacent to the biliary duct, common hepatic artery, portal vein along the superior mesenteric vein, lateral wall of the superior mesenteric artery, pyloric, infrapyloric, subpyloric, proximal mesenteric, coeliac, and posterior and anterior pancreaticoduodenal lymph nodes [1]. During TRIANGLE operation soft tissue between coeliac artery, superior mesenteric artery and mesoportal axis is additionally removed as well as a dissection of the nervous plexus around the superior mesenteric artery of at least 180° is performed [8].

The current German guidelines do not recommend extended lymph node dissection beyond locoregional stations, as no improvement in survival has been observed. Circular arterial dissection has been associated with increased postoperative morbidity, whereas resection of the nervous plexus led to a significant decrease in postoperative quality of life due to diarrhea [1].

In the first descriptive pilot study analyzing 15 patients, Hackert et al. showed good postoperative quality of life in 11 patients without postoperative mortality during the observational period and acceptable postoperative morbidity [3]. Chen and colleagues analyzed safety of the TRIANGLE operation and its impact on recurrence and overall survival in a retrospective cohort analysis of 127 patients, who underwent pancreaticoduodenectomy between January 2017 and April 2023 [9]. After exclusion of patients who received preoperative chemotherapy, died of non-neoplastic causes, or where follow-up data were incomplete, 107 patients were included, 52 underwent TRIANGLE, and 55 underwent standard surgery. There was no significant difference in the duration of surgery, blood loss, length of stay, rate of postoperative complications including postoperative pancreatic fistula, hemorrhage, chyle leak, or proportion of patients receiving at least 6 months of chemotherapy postoperatively. However, a significant reduction in local (37,8 % vs. 16 %) as well as combined local and distant recurrence (26,7 % vs. 12 %) in the TRIANGLE cohort was observed. Additionally, patients who underwent TRIANGLE had significantly longer median recurrence-free and overall survival. TRIANGLE operation proved to be an independent prognostic factor for lower recurrence, both locally and in combination. In a subgroup analysis, the median recurrence-free survival was lower in the subgroup that received TRIANGLE operation and adjuvant chemotherapy than in the group receiving standard operation and adjuvant chemotherapy (30 vs. 16.4 months, p=0.027). Additionally, patients who underwent TRIANGLE operation without adjuvant chemotherapy had a longer recurrence-free survival than those with neither TRIANGLE operation nor adjuvant chemotherapy (24.6 vs. 9.8 months, p=0.013). Chemotherapy regimens were evenly distributed among the groups, with a large majority of gemcitabine-based protocols [9]. However, the study was conducted retrospectively at a single center, and the transferability of the data is limited owing to changes in chemotherapy regimens in recent years [9]. The currently ongoing TRIANGLE Trial aims to overcome these limitations. In this randomized multicenter trial, 270 patients are to be included, and overall survival of pancreatic cancer patients undergoing TRIANGLE operation vs. standard pancreaticoduodenectomy will be analyzed as the primary outcome. Secondary outcomes include oncological parameters, as well as perioperative and patient-reported outcomes [8].

### Autologous patches

Pancreatic resection is characterized by significant postoperative mortality and morbidity, even in high-volume centers [1]. Among the most prevalent complications, postoperative pancreatic fistula (POPF) and post-pancreatectomy hemorrhage (PPH) remain problematic [2], 10]. POPF is associated with increased hospital stay and mortality and may lead to PPH due to arterial erosion [10], whereas PPH, is associated with substantial mortality [2].

Several surgical techniques using autologous patches have been developed to prevent potentially fatal complications [2], 10], 11].

In 2024, Hang et al. performed a meta-analysis of postoperative outcomes after the application of a pedicled ligament or an omental patch [2]. Following systematic literature review, six studies were included for qualitative analysis: one retrospective observational study, three retrospective cohort studies, one retrospective case-control study, and one randomized controlled study [2].

Patch plastic either involved wrapping of the pedicled falciform or teres ligament around the common hepatic artery and the gastroduodenal artery stump or flooring the pedicled ligament flap via fixation on the retroperitoneal tissue. In both cases, the major vessels are separated from the abdominal cavity and thus from abscesses, pancreatic, or bile juice, thereby lowering the risk of erosion. Additionally, compression of injured vessels is surmised to reduce bleeding [2]. Four studies, encompassing a total of 1,140 patients, reported the primary outcome of hepatic or gastroduodenal artery PPH, of which 505 underwent pancreatoduodenectomy with a pedicled ligament patch and 635 without. The incidence of PPH was significantly lower in the patch group than in the controls (2.8 % vs. 7.4 %, OR, 0.41; 95 % CI, 0.22–0.75; p<0.01). Concerning overall PPH, a meta-analysis of 3,035 patients showed that the application of pedicled ligament patches led to a significant reduction in PPH (OR: 0.65; 95 % CI, 0.46–0.93; p=0.02). There were no significant differences in POPF and reoperation rates, morbidity, or mortality between the two groups [2].

However, the meta-analysis was limited due to the high risk of bias, as only one randomized controlled trial (RCT) was included, and the majority of retrospective analyses neglected risk factors for the development of POPF, such as soft pancreatic tissue or small main pancreatic duct diameter [2].

Notably, Welsch and colleagues reported in their RCT in the subgroup of soft pancreatic tissue in the per-protocol analysis PPH rates of 13 % in the control group vs. 4 % in the patch group (OR: 0.26; 95 % CI 0.07 to 0.93; p=0.032) [11].

Concerning the prevention of POPF, a Dutch study analyzed the impact of teres ligament wraps around pancreaticojejunostomy in a prospective cohort [10]. A total of 118 consecutive patients who underwent either open or robotic pancreatoduodenectomy at a single center were included. The first 57 patients did not receive teres ligament wrap, whereas pancreaticojejunostomy was performed in the remaining 61 patients [10].

The authors showed a significant reduction in POPF rates in the teres ligament cohort as opposed to the control cohort (28.1 % vs. 3.3 %, p<0.01) as well as a significantly shorter length of hospital stay in the patch group (11.5d vs. 18.8d, p=0.03) [10].

However, there were no significant differences in PPH rates, the occurrence of biliary or chyle leaks, or mortality. Schipper et al. hypothesized that encapsulation of pancreatic fluids due to the ligament wrap prevented leakage of pancreatic fluids into the abdominal cavity. As a single-center cohort study with a relatively small study population, the trial had several limitations; however, the procedure proved to be safe and technically undemanding [10].

Future multicenter RCTs are needed to provide more robust evidence to guide clinical decision-making and optimize the use of autologous patches in pancreatic resection procedures.

### Robotic pancreatectomy

Pancreaticoduodenectomy (PD) is regarded as one of the most complex abdominal surgeries. Therefore, the introduction of minimally invasive techniques for pancreatic surgery has taken longer than that in other surgical fields. The potential benefits of the minimally invasive technique include less pain, shorter hospital stay, and reduced wound infection rates. However, the clinical outcomes of studies comparing open pancreaticoduodenectomy (OPD) with laparoscopic pancreaticoduodenectomy (LPD) remain controversial [4]. Robotic systems provide new benefits in terms of improved motion range and flexibility and overcome thereby some limitations of the laparoscopic approach [6].

In 2024, a multicenter randomized controlled trial comparing open and robotic pancreaticoduodenectomy and enrolling 164 patients reported a shorter postoperative hospital stay in the robotic group (median 11 vs. 13.5 days; p=0.029). The postoperative 90-day mortality and incidence of severe complications (Dindo-Clavien grade ≥III) were similar between the two groups (p=1.0 and p=0.82) [5].

Tang et al. performed a meta-analysis of randomized controlled trials and propensity-score-matched studies to compare short-term outcomes of robotic and open pancreaticoduodenectomy in 2024 [4]. In total, two randomized controlled trials and 22 propensity score-matched studies were included, with a total of 9,393 patients. Patients in the robotic group had a longer operative time but reduced blood loss, shorter length of hospital stay, lower blood transfusion rate, and fewer wound infections. Furthermore, there were no significant differences in 90-day mortality, overall morbidity, major complications, reoperation, bile leak, POPF, PPH, or delayed gastric emptying. The number of lymph nodes and R0 resections were comparable between the two groups. The authors see robotic pancreaticoduodenectomy as a safe and effective alternative in the future [4].

However, data comparing medium- and long-term outcomes are still lacking and should be the subject of further studies.

Another study by McCarron et al. reported a 10-year experience with robotic pancreaticoduodenectomy with international benchmarks for open pancreaticoduodenectomy [12]. A total of 201 robotic resections were included. In low-risk patients, the outcome was within the benchmark cutoff value. High-risk patients were outside the cut-off values for blood transfusion (26 vs. ≤23 %), overall complications (78 vs. ≤73 %), grade I-II complications (68 vs. ≤62 %), and readmission (22 vs. ≤21 %). The oncologic outcomes were within the cut-off values. In addition, the authors reported a decline in the conversion rate and operative time after 41 robotic cases, whereas the complication rate did not differ over time [12].

The adoption of robotic pancreatic resection has increased over time, and Khachfe et al. compared robotic and laparoscopic pancreaticoduodenectomies with the question of whether the laparoscopic approach is still justified in 2023 [13]. Between 2014 and 2019, 885 robotic and 655 laparoscopic pancreaticoduodenectomies were performed. The robotic group had fewer complications (any complication; p=0.004) and serious complications (p=0.011) [13].

Robotic pancreaticoduodenectomy is an evolving field, and structured training programs are required to learn this complex procedure. Zwart et al. published a study assessing the safety and feasibility of a multicenter training program for robotic pancreaticoduodenectomy in 2022 [7]. The LAELAPS-3 program includes a simulator curriculum, biotissue drills, video training, case observations, and proctoring. In seven centers 275 robotic resections were performed by 15 trained surgeons. The learning curve inflection point for operative time was 22 robotic cases with similar rates of Dindo-Clavien grade ≥III complications in the first and second phase (43.4 vs. 43.8 %; p=0.956) [7].

## Discussion

Although there has been much progress in perioperative management and technique since pancreatic resection was first described in 1909 [14], pancreatic surgery remains one of the most complex procedures with high perioperative mortality and morbidity [1]. Innovative advances are needed to improve patient safety, as well as general, surgical, and oncological outcomes. The TRIANGLE operation, autologous patches, and introduction of robotic surgery in pancreatic surgery target different aspects in this vein: improvement of oncological resection and survival, reduction of postoperative complications, postoperative mortality and morbidity, and postoperative wellbeing. The topics are discussed in the aforementioned order.

### Triangle operation

The TRIANGLE operation constitutes an innovative surgical approach to improve prognosis after pancreatic resection for pancreatic cancer [3]. Current European guidelines do not advocate for extended lymphadenectomy [15]. This recommendation is based on the ISGPS consensus paper, which determined that the extensive resection of lymphatic tissue does not confer survival benefits and is associated with adverse effects such as diarrhea and weight loss [16]. The current German guidelines do not recommend extended lymphadenectomy either [1], referring to a Cochrane Analysis of 2021, which found no difference in survival between standard and extended lymphadenectomy and low-quality evidence of longer operation duration and blood loss. Staerkle et al. concluded that the current evidence did not support or refute the effect of extended lymphadenectomy [17].

### Autologous patches

The data on autologous patches is still not sufficient to recommend patches in general. Many studies are of retrospective character, contain major bias and RCT are scarce. A current meta-analysis reported no difference in POPF, reoperation rates, as well as morbidity and mortality [2]. However, the RCT from Welsch et al. indicated a lower PPH rate in the patch group (4 vs. 13 %) analyzing the subgroup of soft pancreatic tissue [9]. The prospective cohort study from Schipper et al. showed a significant reduction of POPF rates in the teres ligament cohort (3.3 vs. 28.1 %) and a shorter length of hospital stay (11.5 vs. 18.8 days), but there was no difference concerning the PPH rate [8]. Although the available studies are not in line, autologous patches may offer some benefits in reducing POPF and PPH especially in high-risk patients and their use should at least be considered by the attending surgeon.

### Robotic pancreatectomy

Minimally invasive abdominal surgery has prevailed because its benefits include less postoperative pain, shorter duration of hospitalization, and lower incidence of postoperative wound infection. With the introduction of robotic minimally invasive surgery, several limitations of the laparoscopic approach, such as a restricted range of movement, have been overcome [4]. In general, 30–50 % of the cases might be good robotic cases without relevant risk factors. Currently, however, the robotic approach is used in less than 10 % of all pancreaticoduodenectomies [10].

European guidelines allow minimally invasive surgery to potentially reduce morbidity; however, open pancreatic surgery is defined as the standard of care because data on oncological outcomes are lacking [15]. The ESMO guidelines refer to a meta-analysis of two RCTs in which the oncological subgroup, albeit relatively small, showed comparable results [18]. No recommendations have been made concerning robotic surgery.

A recent randomized controlled phase 2b trial conducted at a high-volume center, involving 81 patients, compared open pancreaticoduodenectomy (OPD) with robotic pancreaticoduodenectomy (RPD). The study found that both groups exhibited comparable outcomes in terms of the comprehensive complication index and 90-day mortality rates. However, the incidence of grade B/C pancreas-specific complications, as well as the associated costs and duration of surgery, were significantly higher in the robotic cohort. Notably, more than one in five patients (23 %) required conversion from robotic to open surgery [6].

According to the current German guidelines, distal pancreatectomy may be performed either laparoscopically or robotically. However, these procedures are restricted to being performed within the context of clinical studies or as part of quality assurance measures in specialized centers. No recommendations concerning pancreatic head resection have been made. Furthermore, the recommended minimal volume for pancreatic cases per year is 20 cases in Germany [1]. Thus, a pancreatic center would need around 100 cases to perform 30 robotic resections in appropriate cases per year to reach the minimal recommended case load [1], 10]. Another aspect is the learning curve and the training programs necessary to learn the robotic technique. In the study of Zwart et al. the learning curve inflection point for operative time was 22 cases [12]. These aspects lead to more centralization and the development of high-volume centers as smaller centers cannot provide the necessary cases.

## Conclusions

The TRIANGLE procedure, application of autologous patches and introduction of robotic surgery are innovative approaches in pancreatic surgery.

Overall, this review emphasizes the ongoing need for innovation to improve outcomes in pancreatic surgery, which remains a complex field with high morbidity and mortality. Further studies, especially on the long-term results, are crucial to determine the true value of these emerging techniques.
